# A simulation-based option to assess data-limited fisheries off West African waters

**DOI:** 10.1038/s41598-023-42521-3

**Published:** 2023-09-15

**Authors:** Richard Kindong, Feng Wu, Ousmane Sarr, Jiangfeng Zhu

**Affiliations:** 1 College of Marine Sciences, Shanghai Ocean University, Shanghai, 201306 China; 2 Key Laboratory of Sustainable Exploitation of Oceanic Fisheries Resources, Ministry of Education, Shanghai, 201306 China; 3 Key Laboratory of Oceanic Fisheries Exploration, Ministry of Agriculture and Rural Affairs, Shanghai, 201306 China; 4 National Engineering Research Centre for Oceanic Fisheries, Shanghai Ocean University, Shanghai, China

**Keywords:** Conservation biology, Population dynamics, Marine biology

## Abstract

Most sophisticated stock assessment models often need a large amount of data to assess fish stocks, yet this data is often lacking for most fisheries worldwide, resulting in the increasing demand for data-limited stock assessment methods. To estimate fish stock status, one class of these data-limited methods uses simply catch time series data and, in other instances, life history information or fishery characteristics. These catch-only methods (COMs) built differently are known to make assumptions about changes in fishing effort and may perform differently under various fishing scenarios. As a case study, this paper used European anchovy (*Engraulis encrasicolus*) caught in the northwest African waters, though very economically and ecologically important, but still unassessed. Our study investigated the performance of five COMs under different fishing scenarios using as a reference the life-history information of the European anchovy captured in this region of the Atlantic. Hence, the present study developed a simulation approach to evaluate the performance of the five COMs in inferring the stock biomass status (B/B_MSY_) with consideration of different fishing scenarios under prior information true to anchovy. All five COMs mostly underestimated B/B_MSY_ throughout the simulation period, especially under constant fishing mortality, and in the last five years of the simulation during all fishing scenarios. Overall, these COMs were generally poor classifiers of stock status, however, the state-space COM (SSCOM) generally performed better than the other COMs as it showed possibilities of recovering an overfished stock. When these methods were explored using actual anchovy catch data collected in the northwest African waters, SSCOM yielded results that were deferred from the other COMs. This study being the first to assess this species’ stock in this area using a suite of COMs, presents more insights into the species stock status, and what needs to be considered before scientifically putting in place management measures of the stock in the area.

## Introduction

Despite the presence of comprehensive stock assessments that consider factors such as life history, age, and abundance trends in many stocks located in developed parts of the world^1^, the majority of global stocks remain unevaluated^2^. This can be attributed to several factors, such as the lack of resources for data collection and evaluation. This problem is more pronounced in developing regions and regions with high species diversity but is also seen in developed countries for stocks with small population sizes or low economic value^3,4^. Even more widely, the commitment to the United Nations Sustainable Development Goals (one such aim being the restoration of stocks to Maximum Sustainable Yield levels as specified in Goal 14) implies a need to have a better understanding of the status of the world's stocks. In the United States, Europe, and Australia, where there is a lot of data available in the form of a time series of catches (which includes landings and discards), many new methods have been developed to analyze the “catch-only” family of data-limited fisheries^5–8^.

To provide alternative assessment methods for species with limited data, data-limited assessment models (DLMs) have been developed. DLMs can be broadly classified into length-based and catch-based models based on the information required by the models. Both methods have been used to estimate species stock status and develop feasible fishery management. DLM applications in stock assessment range from developed to developing countries^9–11^. DLMs, according to Zhou et al.^7^, can provide estimates comparable to comprehensive stock assessments. COMs are data-limited stock assessment techniques that rely largely on time series of catch or landings to calculate biomass status (like B/_BMSY_ or depletion) and other key fisheries reference numbers and figures. Some COMs use mechanistic population models, utilizing catch data, potential fishing effort and informative priors on depletion and demographic values^12–14^. Others involve empirical models which are trained on info-rich stocks to predict status with the aid of further information like location, life history or fishery traits^2,9,15^. Most recently, composite approaches have been used to join individual method projections to improve predicted estimates even further^5^.

COMs are broadly classified into four categories by Free et al.^8^: graphical approaches, empirical approaches, mechanistic approaches, and ensemble approaches. Graphical approaches (e.g., stock status plots) have been widely used but criticized for their bias and inaccuracy in inferring stock status^16,17^, whereas empirical and mechanistic approaches have been developed, tested, and applied the most^8,18^. Ensemble approaches combine the strengths of individual COMs to make the most accurate and least biased predictions about stock biomass status^5,8^. We created a simulation framework to test the impact of different fishing scenarios on simulated catch and abundance index data on stock status estimates from five different types of COMs. One empirical method, the Catch-only boosted regression trees (zBRT)^9^; and four mechanistic methods, Catch-MSY methods (CMSY-2013^13^ and an updated catch-MSY (CMSY-2017))^12^, SSCOM State-space catch-only model^14^, and OCOM Optimized catch-only model^19^. These approaches were chosen as candidate models in the present simulation testing because of their relatively reliable performance in previous studies^8,20^ and because their relevant models represent mechanistic and empirical approaches.

Catch data is primarily used in catch-based models to estimate stock status and other reference points for fishery management. Catch data is frequently the most available data for data-poor stocks and can thus be used to provide important information about stock status. For example, the catch has been linked to biomass at the maximum sustainable yield (B_MSY_)^2,9^. Catch data has also been used to estimate population dynamics depletion rates^13,14^. However, it has been suggested that catch is influenced not only by population abundance, but also by fishing effort, catchability, selectivity, and fishery management^8,21,22^. Models based on catch may perform differently under different fishing conditions. Few studies, however, have examined the effects of fishing mortality history on the performance of catch-based models in depth^22^. Catch-based models had different biases under different exploitation rates and for species with different traits, according to Free et al.^8^. As a result, when using catch-based models, these effects should be carefully considered. However, while some studies have examined the effects of fishing history and life history traits on the performance of catch-based models^8,10^, very little research has been conducted on the possible effect of fishing history using catch-based models on small pelagic species in the northeastern Atlantic (species in west African waters).

According to 2022 FAO-Fishery Committee For The Eastern Central Atlantic (FAO-CECAF) report on small pelagic fish off Northwest Africa, the average total catch for the period 1990–2021 has been fluctuating with an average of around 2 million tons, while total catch for the sub-region decreased by 10% from 2020 (2.6 million tons) to 2021 (2.3 million tons), reaching a level that is lower than the average of the last five years (2.6 million tons)^23,24^. Amongst the commercially economic and ecological small pelagic species, we have the European anchovy (*Engraulis encrasicolus*) which is widely distributed in the Eastern Atlantic, Mediterranean, southern Indian Ocean and the Black Seas^23^. This pelagic species typically inhabits shallow waters up to 50 m deep, but can also be found at depths of up to 400 m and usually forms large shoals and migrates, tolerating salinities ranging from 5 to 41%^23^. Anchovy’s spawning occurs multiple times between April and November, with peaks typically occurring during the warmest months^23^. Their growth is rapid, with fish reaching a length of 9–10 cm after one year. First spawning typically occurs at sizes greater than 12–13 cm, and the maximum age of individuals from West Africa is reported to be 3 years^23^. This species primarily feeds on planktonic organisms, including copepods, cirripede and mollusc larvae, and fish eggs and larvae. It is common to reach a maximum size of 20 cm standard length, with most individuals measuring between 12 and 15 cm^23^. They also serve as prey to many important large pelagic species like tunas and tuna-like species. In northwestern African waters, anchovy is mostly harvested in the Moroccan and Mauritanian waters by seiners and pelagic trawlers from Morocco, Mauritania, Russia, Ukraine, and European Union fleets^24^. The population of this species in this region is subject to significant fluctuations due to changes in environmental factors. This species has been evaluated as Least Concern according to the IUCN (International Union for Conservation of Nature and Natural Resources)^23^. However, it is crucial to continue monitoring their population and providing scientific analysis important for the sustainable exploitation of this species in the subregion. According to the FAO-CECAF, the current fishing pressure seems sustainable and can be maintained for future exploitations^24^. However, this statement needs more scientific backing as stock assessments for this species are scarce in this region. Also, maintaining the same fishing pressure as suggested as aforementioned might not be sustainable in the long run, so it is as well important to test different fishing scenarios using different assessment models to make sure that changes in fishing pressure can be captured by models if changes are to happen in the future. Hence in the present study, the development of an assessment framework for anchovy stock in northwest African waters is based on simulation and model performance testing of catch-only methods.

The assessment of European anchovy in the northwest African waters is the primary objective of this study, however, different COMs would first be simulated and tested, and COMs with the least bias and better accuracy identified. Hence, all COMs shall be applied using the actual available data for European anchovy to run assessments; and results shall be presented and discussed. The present study's simulation framework entails applying five catch-only methods to various simulated fishing history scenarios and evaluating their capacity to determine stock biomass status (i.e., B/B_MSY_) using performance metrics. While prior broad modelling studies have confirmed COMs' poor performance, this research may offer an understanding of how various kinds of fishing scenarios can provide biased estimates of stock biomass status from COMs, as well as identify whether different catch data based on the simulated fishing scenarios can be used to test COM performances by comparing the estimated results from COMs for different scenarios to the simulated results. Thus, providing a simulation option for testing catch-only models for fisheries with limited data in West African waters, presenting a plethora of management choices for the assessed stocks leading to sustainable exploitation of such fisheries.

## Results

### Simulation outputs by fishing strategies

**Figure 1:**
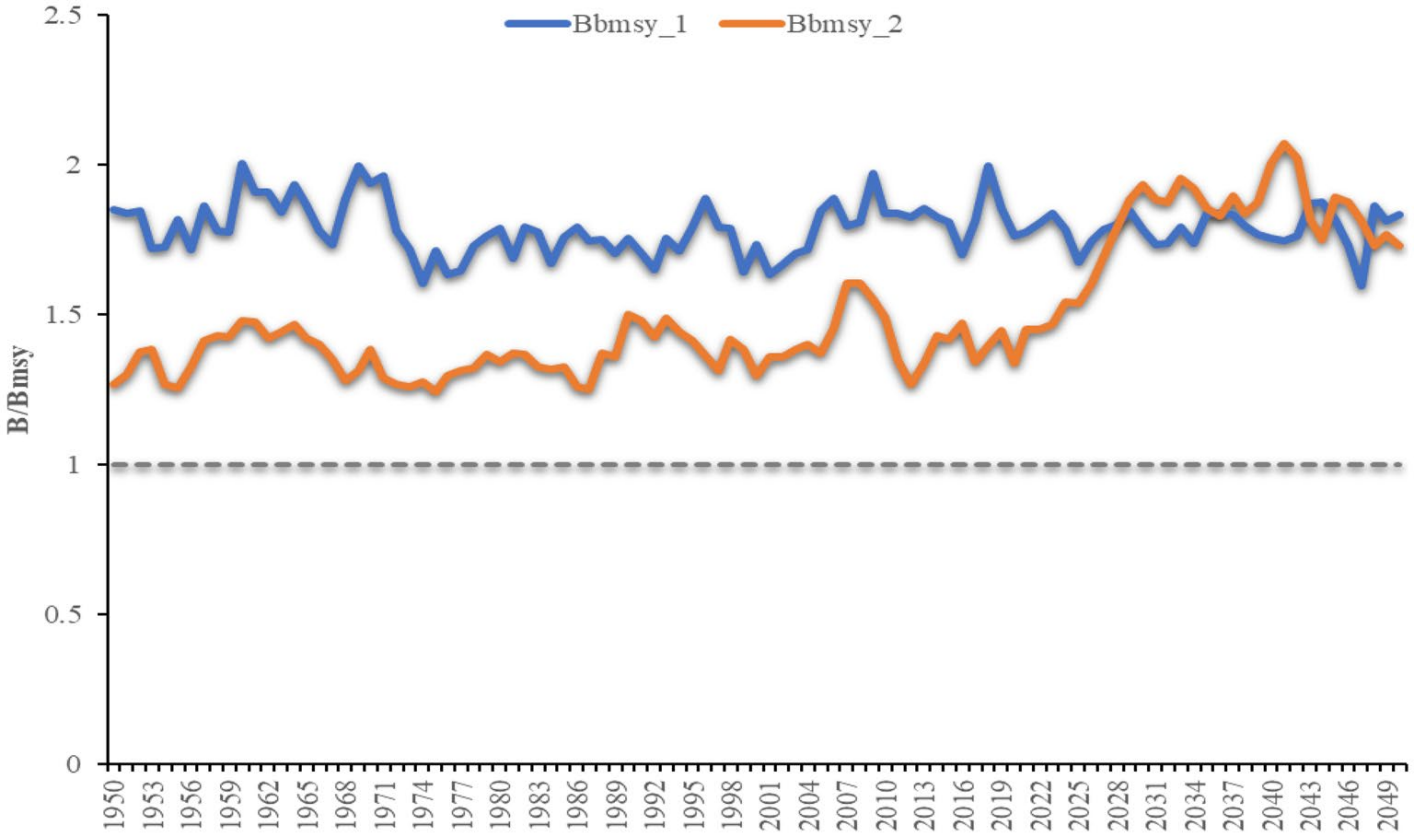
Simulated output results from JABBA for B/B_MSY_ for the two case scenarios. Bbmsy_1 represents the output for scenario 1 and Bbmsy_2 represents the output for scenario 2.

Using the simulated catch and an index of abundance time series from the OMs developed, the biomass at relative B_MSY_ was obtained from JABBA assessments based on these parameters for the two fishing scenarios (Fig. 1). The trends of the simulated biomass were similar till the 75th year when fishing mortality was equal to F_MSY_ for bbmsy_2 (scenario 2), thereby equaling bbmsy_1 (Scenario 1). Scenario 1 represents simulation when F is constant throughout the 100-year simulation period, while Scenario 2 represents simulation when F equals 2*F_MSY_ level during the first 75 years of the simulation (1950–2024) and later permits stock recovery at the MSY level. The simulated biomass for scenario 1 was higher till the 75th year when the biomass for both cases was identical.

### Performance evaluation of the COMs to the simulated true B/B_MSY_ over different time scales

**Figure 2:**
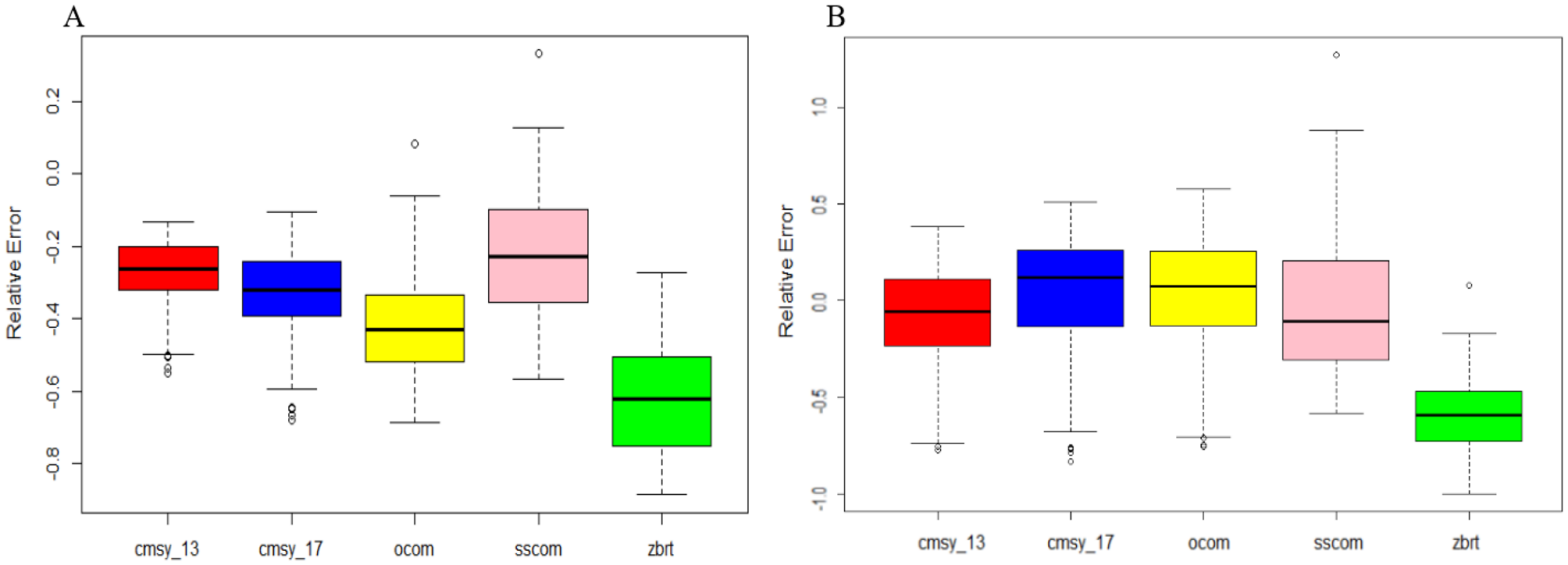
The boxplots indicating the relative errors (RE) between the estimates of the catch-only methods and the simulated true B/B_MSY_. (**A**) Scenario 1 and (**B**) Scenario 2.

During the model fitting and simulation process, all COMs succeeded to estimate B/B_MSY_ in all simulation replicates under both fishing scenarios, except for a few failures during the early stages of simulation for SSCOM under both scenarios. However, the COMs had different performances under different fishing scenarios. For scenario A (constant F), all COMs underestimated B/B_MSY_ with CMSYs and SSCOM performing better (i.e., with RE closer to 0). Whereas for scenario 2 (when F = 2*F_MSY_ the first 75 years of the simulation), all COMs except zBRT performed better and with REs not showing a large significant difference from zero (Fig. 2).

**Figure 3:**
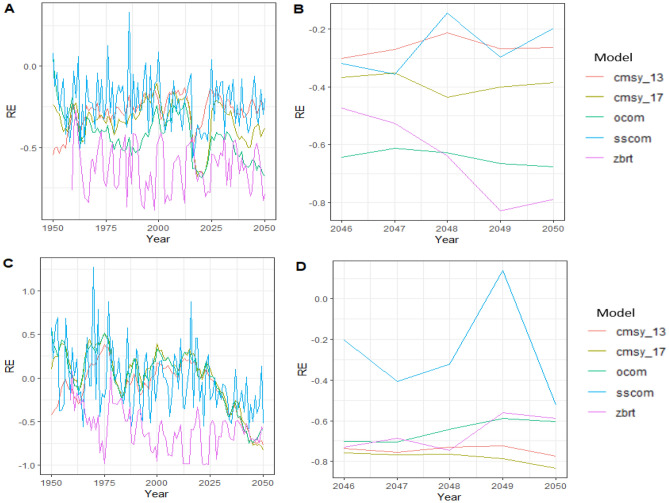
Relative errors trend graph comparing estimated status from COMs to simulated status across all assessment years and the last five recent years for scenario 1 (constant F) and for scenario 2 (F = 2*F_MSY_ level during the first 75 years of the simulation (1950–2024) and later permit stock recovery at MSY level). (**A**) RE trend during all years for scenario 1; (**B**) RE trend during last five years (1946–2050) for scenario 1; (**C**) RE trend during all years for scenario 2; (**D**) RE trend during last five years (1946–2050) for scenario 2. Different lines and colours represent REs for different COMs.

When comparing the performances of COMs between different fishing scenarios, our results showed that COMs generally performed best under scenario 2 (F = 2*F_MSY_; increasing then decreasing fishing), though, most model estimates failed in reflecting biomass recovery when the fishing mortality was decreasing in the last 25 years of the simulation except for SSCOM (Fig. 3C). For scenario 1, when fishing mortality was constant, the COMs generally underperformed by frequently underestimating B/B_MSY_ (Fig. 3A). During the last five years, under the two scenarios of fishing mortality, the COMs generally underestimated B/B_MSY_ (Fig. 3B,D). However, for both scenarios, SSCOM was more robust to different fishing scenarios than the other four COMs. In general, the mechanistic state space COM (SSCOM) was the best predictor of stock status under conditions of constant fishing and varying conditions of excess stock overexploitation and decreasing fishing scenarios. It produced the least biased and close precise estimates of B/B_MSY_ when compared to the B/B_MSY_ of our simulated stock. The SSCOM model was closely followed by the CMSY methods in better predicting B/B_MSY_ stock status, while the empirical method zBRT performed poorly under both scenarios (Fig. 3).

**Figure 4:**
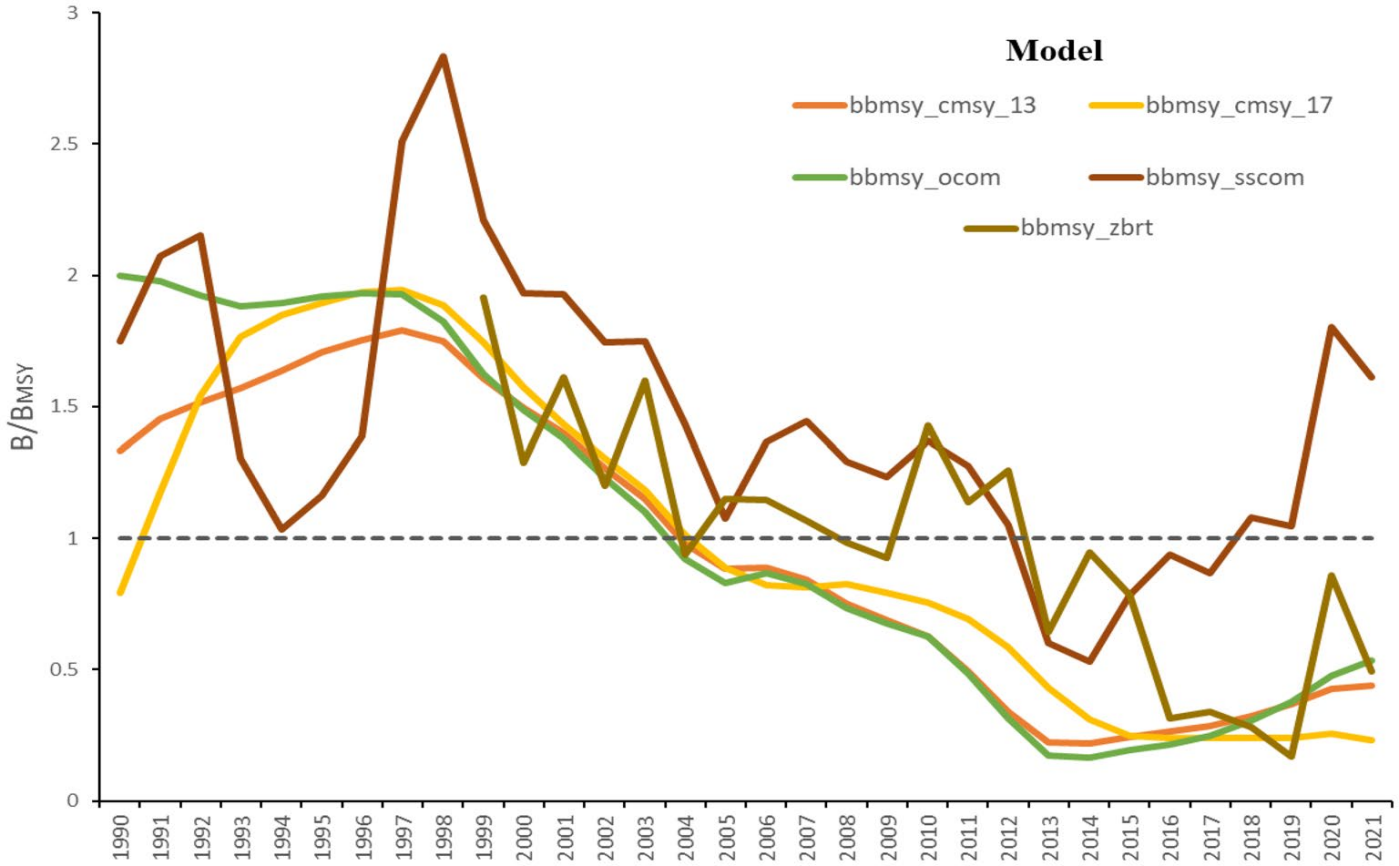
Final assessment results of the European anchovy from COMs using CECAF’s data in the northwest African waters.

Using catch time series data from FAO-CECAF for European anchovy, assessments were run using the five COMs. The biomass trend was not constant throughout the time scale for all models. Early on, most COMs showed healthy stock status for this species, however, towards recent years, four out of five models indicated overfished status for European anchovy except for SSCOM (B/B_MSY_ > 1) (Fig. 4). All COMs had trends indicating a healthy state of the stock up till 2003 when the tendency changed from healthy to overfished with OCOM and CMSY methods not indicating possibilities of stock recovery till recent years. zBRT recovered after 2003 only for the biomass to drop below 1 after 10 years, and stayed like that till recent years. SSCOM indicated a healthy stock state from 1990 till 2012 when the biomass dropped below 1 but recovered in 2017 till recent years of the fishing period. As observed during the simulation phase of the COMs, SSCOM provided a better estimate, close to the simulated true stock status. Therefore, for the present case of European anchovy stock, SSCOM may be taken more seriously when compared to the other four COMs.

## Discussions

Given that many fisheries in the world are still unassessed, the development and the use of many data-limited fisheries have been growing and employed to assess these fisheries. These types of models be they length-based or catch-based are uncertain, although data-rich models present more reliable results, they are less uncertain than these data-limited models. Therefore, the need to always test the performances of data-limited methods before applying them to assess fisheries for management. The present study developed OMs based on European anchovy species captured from the northwest African waters to simulate catch and an index of abundance time series data for this stock. The output from the developed OMs was run in the JABBA model to obtain the simulated biomass status of European anchovy stock defined as the ratio of the biomass relative to the biomass at maximum sustainable yield: B/B_MSY_. Based on this parameter, the simulation stock using JABBA under the two tested fisheries scenarios was considered to be in a healthy state (not-overfished and no-overfishing, Fig. S1). The COMs tested in the present study tended to underestimate the biomass status in all fishing scenarios when compared to the simulated biomass status. SSCOM was the only COM that presented a biomass status with the lowest bias and close precision to the simulated stock biomass. Moreover, when these COMs were used to run the assessment of European anchovy using the FAO-CECAF data, all COMs reported that the stock might be overfished, except for SSCOM which reported a healthy stock status, similar to the results when the stock was assessed using simulated data with the species’ life-history parameters.

The simulation carried out in this study aimed to test how efficient COMs would explore stock biomass status under different fishing scenarios affecting various catch times series. This study showed how influential fishing changes can deter results presented by COMs. As observed from our results after simulations, at constant fishing mortality, COMs showed large differences in the estimation of the stock biomass status when compared to the biomass estimated through stock simulated stock. For the scenario when fishing mortality was set twice the F_MSY_ for the first 75 years of simulation, biomass results of OCOM, SSCOM and the two CMSY methods did not show great significant differences to the true biomass estimate from simulation stock. On both occasions, the mechanistic catch-only method, SSCOM presented the least difference in biomass results to the biomass of the simulated stock when compared to the other four COMs. Whereas, the empirical catch-only method, zBRT showed the least reliable estimates of biomass when compared to the biomass of the simulated stock. These simulations showed that for a stock under constant fishing mortality or undergoing excessive fishing, all five COMs would not easily replicate biomass levels when compared to simulated stock results. However, if faced with a choice to select one method amongst the COMs tested here, SSCOM would be the better candidate, since it performed much better than the other COMs, especially for scenario 2 (F = 2*F_MSY_) over most parts of the entire time series (RE ~ 0). With these findings, the present result shows the importance of testing a suite of COMs under various fishing scenarios for species with very little information in areas of high fishing intensity but very little research background that ought to provide management of fishery resources.

Furthermore, as observed in the present study for the two main anchovy simulated scenarios, the values of r and K for COMs in the first scenario fell within the CI of estimates from JABBA run 1 (Supplementary Table S1). Whereas for scenario 2, values from COMs were overestimated as compared to the JABBA run. This might be because COMs couldn’t fit the data well in changing fishing dynamics as was observed in this scenario. Moreover, most COMs such as CMSY, OCOM… used in the present study are based on the Schaefer dynamics equation, whereas the JABBA model used in the present study was run based on the Pella-Tomilson model, this might as well be a contributing factor for the differences observed in the posterior estimations of r and K. And finally, differences in COMs performances could also be linked to how models were built and their configurations.

Using this global knowledge of COMs capabilities observed during simulation phases, we undertook to run the assessment of the European anchovy captured in the northeast Atlantic (northwest African waters) using these COMs to indicate what might be the state of this stock. This study being the first to assess this species’ stock in this area using a suite of COMs would present more insights into the species' stock status and what needs to be accounted for before scientifically putting in place management measures for the stock in the area. Using catch data presented by FAO-CECAF from 1990 to 2021 for European anchovy, including also reliable life history information of the species, we used all five COMs to run assessments. All COMs could fit well the data presented, and results indicated that COMs presented healthy stock status for anchovy during the first half of the catch time series. However, biomass levels dropped below 1 drastically for OCOM and both CMSY approaches after 2003, and did not show the stock recovering till 2021. From the catch year 2003, zBRT dropped twice below 1 and never recovered, whereas, SSCOM indicated biomass dropping once in 2012 and finally revered showing biomass level above 1. Therefore, only SSCOM amongst the four COMs could successfully capture stock recovery, thereby correctly identifying a sustainable status (i.e., B/B_MSY_ > 1) over most parts of the entire time series for this stock in northwest African waters.

The present study identified that SSCOM had the lowest RE values as compared to the other four COMs, followed by the two CMSY methods. Moreover, SSCOM was more stable as compared to other COMs when observing both fishing scenarios tested here. For scenario 2, after fishing for the first 75 years of the simulation, all COMs indicated REs dropping farther from 0, however, SSCOM was the only model with REs revolving around 0 during the last 25 years of the simulation. Other studies equally tested the performances of COMs and had similar conclusions indicating that these methods are not only influenced by fisheries changes but also fish size selectivity, life history strategies and catch misreporting^8,10,13,18,21,25–29^. Although SSCOM showed better simulation results than the other four COMs, it still has its limitations and must be applied with caution for management. For example, Thorson et al.^14^ pointed out that SSCOM is highly dependent on the catch time series and the assessed biomass may be highly correlated with the catch but not with the true biomass dynamics. Though in our study the anchovy catch time series was less correlated to our assessed biomass.

It is clear that the first step in successful fisheries management is estimating stock status. In the FAO-CECAF management region, stock advice is given against agreed reference points^30^. For European anchovy, using length composition data collected for this species, a Length Cohort Analysis^31^ was applied to estimate the current fishing mortality level (F) and the relative exploitation pattern of the fishery over the last few years. And, a length-based Yield per Recruit Analysis^32^ was then run on these estimates, to assess the status of the stock in relation to the biological reference points Fmax and F_0.1_^24^. According to these analyses, anchovy was considered to be moving towards full exploitation level, meaning the fishery operates within the limits of sustainability, and the current fishing pressure seems sustainable and may be maintained. Given that data-limited methods are mainly categorized as length-based and catch-based, there is a need to assess anchovy stock using catch time series data in catch-based models to corroborate results in the FAO-CECAF report. Hence, the implementation of the COMs used in the present work presented different stock status results for the anchovy population in the Northeast Atlantic. The COM with the better bias and precision as observed in the simulation, SSCOM indicated a stock status that recovered from an overfished state for anchovy. This result aligns with results from the length-based Yield per Recruit Analysis used for the same species in the northwest African waters. However, determinations derived from data-limited assessments cannot entirely guarantee proper and sustainable management, therefore, must be applied with caution. The latter phrase can be corroborated by research done on European anchovy in the Bay of Biscay and the Iberian Coast Ecoregion by Cousido-Rocha et al.^48^ that does not recommend the use of length-based data-limited methods, and that their application if necessary should be done with caution. This conclusion was based on their work which compared data-limited methods to data-rich methods applied to European anchovy in the Bay of Biscay and the Iberian peninsula, showing distinct results to the data-rich assessment approach used in the ecoregion.

When used to guide fishery management, existing COMs have limited accuracy and a high likelihood of providing misleading information^25^. Therefore, the development of COMs should not be an excuse for stakeholders and fisheries managers to set up schemes for the continuous collection of data useful to perform more solid and complete assessments. While data-limited models could provide a quick assessment for management decisions, it should be noted that our use of these methods involved simple hypotheses and frequently provided biased estimates of fishery status^8,26^. Because catch-based models are susceptible to fishing history and strategy^25^, as demonstrated in this work, we propose that catch-only approaches be used as a temporary stepping stone while data (e.g., size or age composition, and valid abundance indices data) is acquired to allow for the adoption of more reliable methods.

Our findings corroborate the notion that a species' life history features not only directly impact the prediction of COMs^8,26^, but also correlate to fishing situations in terms of the effect on the performance of data-limited approaches. However, despite having access to considerable data, previous research revealed that all catch-based models already in use experienced difficulties in producing credible findings that corresponded with standard stock assessment techniques^8^. This suggests that catch-based models should be utilized with caution in the future. Therefore, selecting a COM for management as is the case for European anchovy in our study should be done with caution while striving to update fisheries and biological data for use in traditional assessment models.

## Materials and methods

### Methodology overview

This study used a generic operating model (OM) based on an age-structured model to simulate population dynamics and generate catch and an index of abundance times series^33^. The catch and the index of abundance data generated by this OM, coupled with life history information of European anchovy in the northwest African region were used to run the European anchovy assessment implemented using a Bayesian state-space surplus production model framework called JABBA (www.github.com/jabbamodel/JABBA)^34^.

JABBA was used as a simulation tool to provide a measure of determining the stock status: biomass relative to the biomass at maximum sustainable yield, B/B_MSY_). A suite of catch-only methods (COMs) was later used to provide a stock status (B/B_MSY_) using the simulated catch data from the OM. Later, a simulation framework to compare the true values (from JABBA) and estimated values (from COMs) of the stock status (i.e., B/B_MSY_) was developed. We simulated a 100-year population dynamics time series with a constant fishing scenario throughout these times scale and a 75-year fishing period at fishing mortality twice the maximum sustainable yield (2*F_MSY_) in the 100-years simulation using the OM. And this procedure was repeated 500 times for each case scenario. The COMs with an estimated stock status close to the true value obtained in the simulation tool (JABBA) were deemed fit enough to assess the stock status of the species concerned. Hence, these COMs were also applied to determine the stock status of European anchovy using catch data present in the FAO-CECAF database.

### Operation model for simulating population dynamics, catch time series, an index of abundance times series and simulated stock status

As aforementioned, the OM used in this study to model the European anchovy population in the northwest African waters followed an age-structured format as detailed in Cousido-Rocha et al.^33^. The population modeling function in the OM is divided into 4 blocks notably: general information about the population to simulate; biological parameters of the population; fishing parameters of the population; and parameters of the spawning stock-recruitment relationship. A detailed process for this population modeling can be found in Marta et al.^33^. The sampling data from the OM mimics the collection of fishery-dependent data and research surveys. These OM samples catch numbers for each year, age and iteration of the simulations. The catch number for year t and age *i* (*C*_*N,Sit*_) is generated from a log-normal distribution centre in *C*_*Nit*_ and variability is determined for the corresponding *CV*_*CN*_. Also, the index of abundance for each year, age and iteration of the simulations are estimated by the OM. The abundance index (IA) for year *t* and age *i* is estimated from the equation:

$$IA_{it} = q_{Ait} * N^{\gamma }_{it} ,$$

where q_A*it*_ is the catchability coefficient, *γ* = *gamma* is the density-dependent parameter, and *N*_*it*_ is the abundance for year *t* and age *i* (stock numbers matrix). Given that we considered a *CV*_*A*_ different than 0 the abundance index for year *t* and age *i* is generated from a log-normal distribution in *IA*_*it*_ and variability determined from the corresponding coefficient of variation *CV*_*A*_. Details on how the catch and abundance index times series are estimated in this OM can be seen in Marta et al.^33^.

To obtain the measure of the stock status (i.e., B/B_MSY_) important for comparison with COMs, JABBA^34^ was used to determine this parameter using the catch and index of abundance times series data mimicked by the OM. JABBA is a widely used stock assessment model in tuna Regional Fisheries Management Organizations (tRFMOs) to make management decisions on many data-rich species^35,36^. All models were implemented using a Pella and Tomlinson production function in JABBA. The prior environmental carrying capacity K was assumed to be 10 times the maximum catch of anchovy (in 2003 the maximum catch was 179,854 tons, ~ 2,000,000 t). For K*,* a lognormal distribution was implemented using JABBA “range” option; lower and upper values ranged from 50,000 t to 2,000,000 t to allow flexibility in the analysis^34^. For *r*, we developed a prior distribution range (0.5, 2) with an associated shape parameter of a Pella-Tomlinson production function from an Age-Structured Equilibrium Model (ASEM) approach with Monte-Carlo simulations^37^. All scenarios had the same initial depletion prior (*φ* = *B*1950*/K*) defined by a beta distribution with mean = 0.93 and CV of 2%. All catchability parameters were formulated as uninformative uniform priors. The process error of log(*By*) in year *y* for all scenarios was formulated as an uninformative uniform prior and was defined by an inverse-gamma distribution with a shape parameter equal to 0.001 and a rate parameter equal to 0.001. A fixed observation error value of 0.01 was considered in the analysis. JABBA is implemented in R (R Development Core Team, https://www.r-project.org/)^38^ with JAGS interface^39^ to estimate the Bayesian posterior distributions of all quantities of interest using a Markov Chains Monte Carlo (MCMC) simulation. The JAGS model is executed from R using the wrapper function jags() from the library r2jags^40^, which depends on rjags R package. In this study, three MCMC chains were used. Each model was run for 20,000 iterations, sampled with a burn-in period of 5,000 for each chain and thinning rate of five iterations.

### Simulation scenarios

In the simulation (OM), natural mortality was assumed constant and independent of size and age, maturity at length was based on a logistic function, a von Bertalanffy growth function was used to model individual growth, and the stock-recruitment relationship was modeled using the Beverton-Holt model. Two fleets (one commercial fishery and one survey) with time-invariant double normal selectivity patterns were assumed to harvest in a single area. A minor random observation error was included in the form of a standard error of logarithm of catch, which was set to 0.005 in the OM and was assumed not to affect the simulation results. Parameters of life history and fishery traits were collected from various sources of literature for European anchovy mostly from FAO-CECAF (2022)^24^ (Table 1).

**Table 1:** Main life history and fishery traits for European anchovy serving as input parameters in the OM.

Parameter	Symbols	Value	References
Natural mortality (year^−1^)	M	1.35	FAO-CECAF (2022)^24^
Length at infinity (cm)	Linf	17	
Growth rate (year^−1^)	*k*	1.39	
Length–weight scaling (kg cm^−1^)	a	0.0041	
Allometric factor (–)	b	3.1818	
Age at 50% maturity (cm)	A50mat	1	FishBase (2023)^41^
Population size at first age and year	N0	200,000	Assumed
Minimum and maximum age corresponding to fishing mortality	minFage	1	FAO-CECAF (2022)^24^
	maxFage	3	
Age range used		0–5	
Coefficient of variation of M	CV_M	0.15	Assumed
Coefficient of variation of a50_Mat	CV_a50_Mat	0.15	Assumed
Sigmoid’s midpoint of the logistic function	a50_Sel	1.5	Assumed
Coefficient of variation of logistic selectivity	CV_SEL	0.15	Assumed

**Figure 5:**
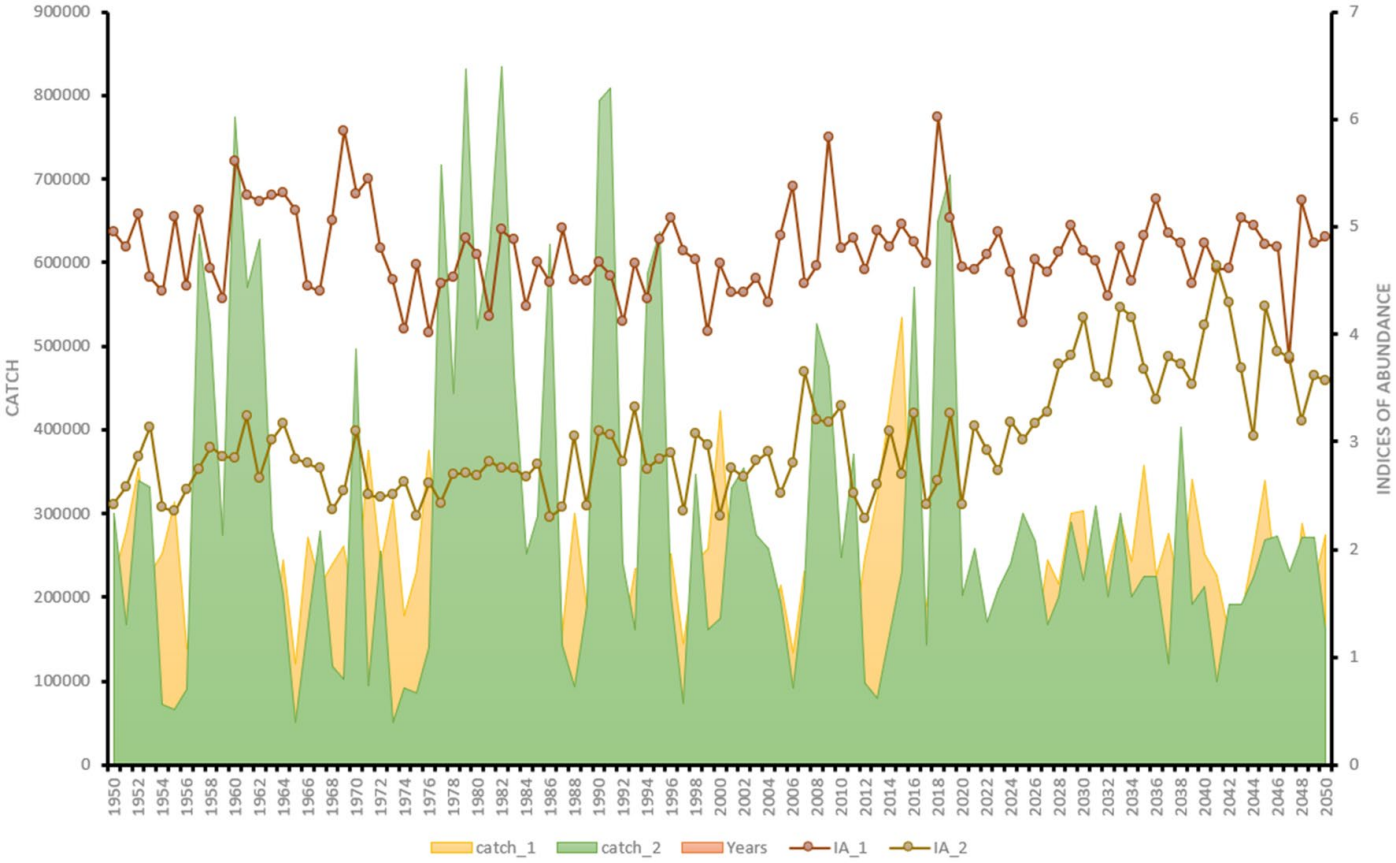
The simulated catch (tons) and index of abundance time series based on two fishing scenarios. Scenario 1: catch_1 and IA_1 represent a simulated catch and an index of abundance when F is constant throughout the 100-year simulation period; Scenario 2: catch_2 and IA_2 represent a simulated catch and an index of abundance when F equals 2*F_MSY_ level during the first 75-years of the simulation (1950–2024) and later permit stock recovery at MSY level.

Our main simulation focus was to see whether catches from different fishing scenarios over different catch time series affect the performance of catch-only models. So, in this study, we simulated population dynamics for two scenarios, including combinations of two fishing cases when fishing mortality was constant at all times of exploitation throughout the 100-year simulation; and when fishing mortality was twice the F_MSY_ level the first 75 years of the simulation and to allow stock recovery at MSY level in the last 25 years (Fig. 5). After constructing the OMs for both cases for this species, we compared the results of B/B_MSY_ for both cases when run separately in JABBA.

### Estimation models (COMs)

For the species under each scenario, five catch-based assessment models were applied to the simulated catch data to evaluate model performance. The parameters used in the catch-based models for anchovy are listed in Table 1. The analyses were conducted by the “datalimited”^42^ and “datalimited2” packages^43^ implemented in the R program (version 4.1.3)^38^.

#### Catch-MSY methods (Catch-MSY, CMSY-2013 and CMSY-2017)

Catch-MSY methods^12,13^ are widely used catch-only stock reduction analyses. In the Schaefer model, stock reduction analyses reconstruct historical abundance and exploitation rates by simulating biomass trajectories that could produce the observed catch time series given informative priors on initial and final year depletion and stock dynamics like carrying capacity, K, or intrinsic growth rate, r. The very first catch-MSY method (cMSY-2013)^13^; sets priors for r and K according to the highest catch (e.g., between Cmax and 10*Cmax), and initial and final year depletion based on the ratio of initial and final year catch to the highest catch. The catch-MSY method takes into account 'viable' pairs of r and K, which do not lead to extinction or year depletion beyond the prior. It then calculates the MSY with the geometric mean of those viable pairs. Rosenberg et al.^20^ modified it so that it could generate biomass trends from all suitable r-K pairs, finding an estimation of B/B_MSY_ from the median trend.

Froese et al.^12^ further developed the CMSY-2017, offering calculations for biomass, exploitation rates, as well as MSY and other important reference points. Additionally, they also included a process to pinpoint probable r-K pairs and tackle the tendency for production models to overestimate productivity when dealing with very low stock sizes. The catch-MSY methods have been applied in several assessments across the globe such as Costello et al.'s^44^ global analysis, Froese et al.'s^45^ regional fisheries assessment, Winker et al.'s^46^ Atlantic shortfin mako shark report, Kindong et al.’s^29^ Atlantic Blue shark assessment, just to name a few. The CMSY-13 required six types of input data, including catch time series, resilience (r), natural mortality (M), carrying capacity (K), and the range of the depletion rate for the first and the final year. We determined that the priors of r and K were from uniform distributions, and we used the Bernoulli distribution as the likelihood function for viable r–K pairs. To obtain enough pairs of the viable r–K combinations (those with which the population would not collapse or be over the carrying capacity, as defined in Martell and Froese^13^), we assumed the maximum value of K as 10 times the maximum catch and the minimum value of r as 0.4. The depletion rate was set as medium depletion (0.2–0.6).

#### Optimized catch-only model (OCOM)

The OCOM model implements a stock reduction analysis, where priors for intrinsic growth rate (r) and final year depletion derived from natural mortality and saturation estimated by zBRT are taken into account. It is based on the Schaefer biomass dynamics model and an algorithm for determining feasible parameter combinations, which allows for the estimation of biological parameters like r, K, and annual biomass as well as management parameters such as MSY, B_MSY_, or F_MSY_. Different from catch-MSY stock reduction analyses with its stochastic approach, OCOM utilises an optimization technique and a more informed depletion prior. The efficacy of the method was checked by Zhou et al.^19^ through 14 Australian fish stocks assessed using Stock Synthesis^47^: it yielded estimates similar to those obtained through full assessments. In the present study, we defined similar prior distribution for the model parameters same as the other models.

#### State-space catch-only model (SSCOM)

Thorson et al.^14^ define the state-space catch-only model (SSCOM) as a hierarchical model based on a coupled harvest-dynamics model. Based on a catch time series and priors on r, the maximum rate of increase of fishing effort, and the amplitude of various kinds of stochasticity, SSCOM estimates unobserved dynamics in both fishing effort and the fished population. Thorson et al.^14^ validated the model through simulation testing and application to eight evaluated US West Coast groundfish populations. For the application of SSCOM in the present study, we defined the prior distribution for the model parameters the same as the previous models. Since the SSCOM considers the stochasticity of the population dynamics, effort dynamics, and catch efficiency using a Bayesian state-space framework, we also defined the process errors of effort, biomass, and catchability.

#### Catch-only boosted regression tree model (zBRT)

By training boosted regression tree models on assessed data-rich stocks in the RAM Legacy Stock Assessment Database^1^, Zhou et al.^9^ developed an empirical catch-only boosted regression tree method (zBRT) to estimate stock saturation (defined as B/K). zBRT employs factors such as the whole catch time series, the subseries before and after the maximum catch, and recent years as its most important predictors. It has been demonstrated that zBRT outperforms other methods like modified panel regression models and earlier catch-based classification approaches. In addition, it only needs a catch time series to determine biomass status (which is estimated as a double saturation value). Because depletion levels (1-saturation) are frequently used in DLMs, this model can provide a more precise prior for these models. Finally, in various studies concerning Stock Assessment Modelling, zBRT was rigorously examined or used to construct ensemble COMs^8,9,11^.

### Performance metrics

In order to examine the performance of catch-only models in estimating stock status under different fishing scenarios, we calculated the relative error (RE) between the model estimated and the simulated stock biomass (B) proportional to the B_MSY_ (B/B_MSY_). We used RE to quantify both bias and precision as a measure of uncertainty for all time scales and the last 5 years^20^. RE is a dimensionless value, which gives the proportional difference between the estimate and the true value, and is estimated thus:

$${\text{RE }} = \left( {\hat{\theta } \, {-} \, \theta } \right)/\theta$$

where $\hat{\theta }$ is the estimated value from the catch-only model and *θ* is the B/B_MSY_ value calculated from the simulated stock by JABBA. A RE value greater than zero indicates that the estimated status is greater than the true status (i.e. the stock is predicted to be healthier than it is). Equally, a RE less than zero indicates an estimated status lower than the true value.

#### Time period (last 5 years versus entire time series)

The computation of Relative Error (RE) was undertaken for the complete duration of the time series and the recent 5 years, specifically, from 2046 to 2050. To achieve a comprehensive evaluation of performance, it is essential to perform both analyses, which encompass the entire time series, including its initial years; such calculations reflect the model's assumptions at the beginning of the time series, such as those regarding initial depletion and periodicity^20^. The analysis considering solely the recent five years presents a momentary evaluation of the current performance distal to the initial conditions. The recent five years are noteworthy for their alignment with management time scales as it provides insights pertinent to managerial recommendations. Where discrepancies were observed in the metric when computed across the two time periods, a thorough visual examination was conducted to elucidate the causal factors behind the fluctuation in performance outcomes attributable to time series influences.

### Assessment of European anchovy in the northwest African waters

**Figure 6:**
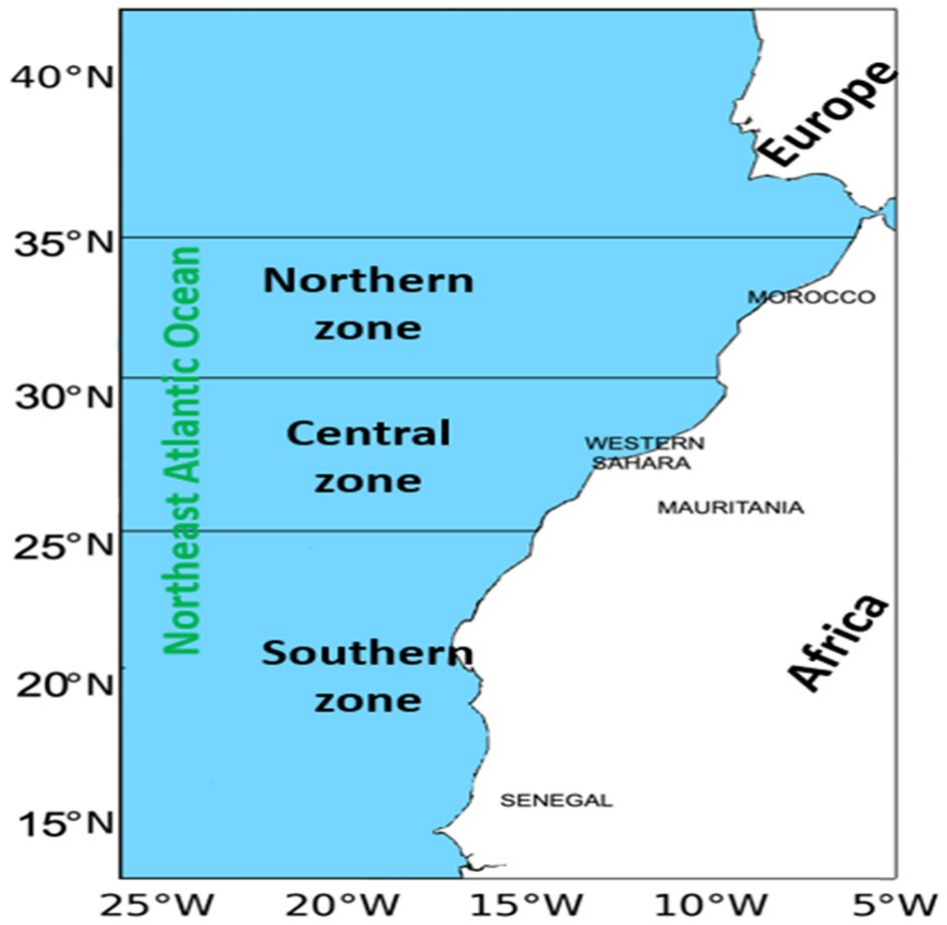
FAO-CECAF Northwest African Area presented according to the zones where European anchovy catches were recorded. The R statistical program (version 4.1.3, https://www.r-project.org/)^38^ was used to develop the map and Microsoft Office PowerPoint 2016 was used to edit the map.

Once the performances of all COM(s) were tested for bias and precision (RE ~ 0), the models were later used to assess the European anchovy in the northwest African waters using actual catch data from CECAF (Supplementary Fig. S2). Respective outputs from the COMs were presented and discussed, with emphasis laid on the COM with RE close to the simulated stock status. The actual catch data used to assess this anchovy stock was gotten in the region between the southern border of Senegal and the northern Atlantic border of Morocco (Fig. 6). The total catch of anchovy (*Engraulis encrasicolus*) in 2021 was around 49,000 tons, indicating a significant increase from 20,000 tons in 2019 (Supplementary Fig. S2). The long-term average catch of the species (1990–2021) is around 79,000 tons, and the average for the last five years (2017–2021) is 33,000 tons. Most harvests for anchovy in the northwest African waters occur in the Moroccan and Mauritanian waters (Fig. 6) using fishing gears such as coastal purse seiners and semi-pelagic trawlers.

### Supplementary Information

Supplementary Information.

## Data Availability

Data is available upon request from the corresponding author.
