# Supplementary material for: A simulation-based option to assess data-limited fisheries off West African waters
Source: Sci Rep. 2023 Sep 15;13:15290. doi: 10.1038/s41598-023-42521-3 (PMC10504299; doi:10.1038/s41598-023-42521-3)
Supplement: Supplementary file 1 — Supplementary Information. [file 41598_2023_42521_MOESM1_ESM.docx]

A simulation-based option to assess data-limited fisheries off West African waters

Richard Kindong^1,2,3,4*^ (kindong_richard@yahoo.com); Feng Wu^1,2,3,4^ (fwu@shou.edu.cn); Ousmane Sarr^1^ (sarrousm99@gmail.com); Jiangfeng Zhu^1,2,3,4*^ jfzhu@shou.edu.cn)

^1^ College of Marine Sciences, Shanghai Ocean University, Shanghai 201306, China

^2^ Key Laboratory of Sustainable Exploitation of Oceanic Fisheries Resources, Ministry of Education, Shanghai 201306, China

^3^ Key Laboratory of Oceanic Fisheries Exploration, Ministry of Agriculture and Rural Affairs, Shanghai 201306, China

^4^ National Engineering Research Centre for Oceanic Fisheries, Shanghai Ocean University, Shanghai, China


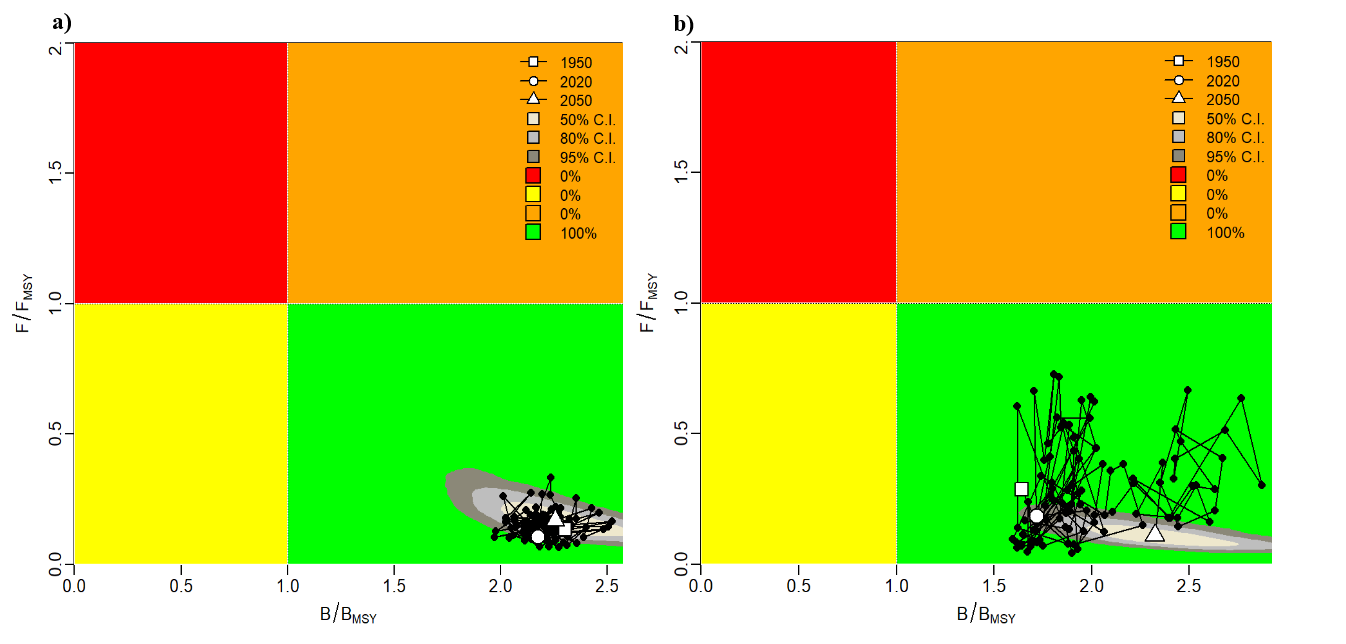


**Supplementary Figure S1**: Kobe Output results from simulated stock scenarios using JABBA

**Supplementary Figure S2**: Actual catch data of European anchovy in the northwest African waters from CECAF

**Supplementary Table S1**: Parameters estimates for each model used

| **Scenario 1** | **Models** | **B/B_MSY_** | **B_MSY_** | **r** | **K** | **F/F_MSY_** | **F_MSY_** |
| --- | --- | --- | --- | --- | --- | --- | --- |
|  | **SSCOM** | 1.47 | 323965.6 |  | 647931.19 |  |  |
|  | **CMSY13** | 1.36 | 720664.3 | 0.76 | 1441328.5 | 0.28 | 0.38 |
|  | **CMSY 17** | 1.12 | 471500 | 1.1 | 943000 | 0.94 | 0.55 |
|  | **OCOM** | 0.59 | 824434.8 | 0.59 | 1648869.673 | 1.88 | 0.29 |
|  | **zBRT** | 0.38 |  |  |  |  |  |
|  | **JABBA_1** | 2.25 | 1219951 | 0.69  (0.35-1.07) | 3072529  (1305259-4548072) | 0.16 | 0.59 |
| **Scenario 2** |  |  |  |  |  |  |  |
|  |  | **B/B_MSY_** | **B_MSY_** | **r** | **K** | **F/F_MSY_** | **F_MSY_** |
|  | **SSCOM** | 0.82 | 2280532 |  | 4561064.08 |  |  |
|  | **CMSY13** | 0.38 | 2460729 | 0.41 | 4921458 | 0.81 | 0.20 |
|  | **CMSY 17** | 0.29 | 948000 | 1.27 | 1896000 | 1.58 | 0.63 |
|  | **OCOM** | 0.68 | 1730660 | 0.60 | 3461320.7 | 0.44 | 0.30 |
|  | **zBRT** | 0.71 |  |  |  |  |  |
|  | **JABBA_2** | 1.73 | 581703.7 | 0.13  (0.06-0.27) | 1475727  (842273.6-3159274) | 0.04 | 0.11 |
